# Case of Poisoning by Prussic Acid

**Published:** 1845-09

**Authors:** Thomas Leithead

**Affiliations:** Warkworth


*Case of Poisoning by Prussic Acid.* By Thomas Leithead, Esq., Warkworth.—On the morning of the 28th ult., a few minutes after 12 A. M., I was called to see Jane H——, aged 17, servant to Mr. Turner, a chemist in the village by a messenger, who stated she had "taken something," and I must make haste. I reached the house within three minutes of the call, and on entering the bed-room found Mrs. Turner supporting the patient on the bed, whilst Mr. Turner was bathing her head with cold water. The countenance had a ghastly appearance; the eyelids nearly shut; respiration protracted and slow; there was no stertor, no convulsions, nor had any been observed. On raising the eyelids, the pupils appeared dilated and insensible; pulse gone at the wrist; nor could I detect any action of the heart. I sent down to Mr. T's shop for some ammonia, but she was dead before I received it. On applying my nostrils to her mouth, I thought I found the smell of prussic acid; Mr. Turner could not. On the following day, fourteen hours after death, on entering the room with the jury, I smelt the acid immediately; liquid oozing from the mouth and nostrils smelt strongly of it. The fluid had run over the bed, and smelt pungently of the acid; the back was much discolored, and the face of a livid hue. The coroner did not consider a *post-mortem* examination necessary.

The body was removed from the bed, and in turning up the feather bed, a prussic acid bottle, with the stopper in, was found between it and the mattress, near the centre; it contained about eight drops of the acid. The girl's mistress states that about twenty minutes after the girl had left the room, she was proceeding up stairs to bed, when, in passing the girl's room door, she heard a moaning noise, she entered the room, and found her lying in bed, with her clothes on, and the bed-clothes drawn up to her face, apparently "gasping for breath." She instantly gave the alarm, and I was sent for.

The evidence adduced proved—as far as *could* be proved—that she had swallowed an ounce of the acid, re-corked the phial, thrust it to full arm's length between the feather bed and the mattress, got into bed, and then drawn the clothes over her body; and there appeared to have been no convulsions.—*London Lancet.*

